# Antibacterial Activity and Cytotoxicity of the Novel Bacteriocin Pkmh

**DOI:** 10.3390/ijms25179153

**Published:** 2024-08-23

**Authors:** Yu Wang, Xiaojia Fu, Yue Wang, Jun Wang, Lingcong Kong, Haiyong Guo

**Affiliations:** 1 College of Life Science, Jilin Normal University, Siping 136000, China; yu231147@jlnu.edu.cn (Y.W.); fuxiaojia2024@163.com (X.F.); 15134330583@163.com (Y.W.); 17856583478@163.com (J.W.); 2 College of Veterinary Medicine, Jilin Agricultural University, Changchun 130118, China

**Keywords:** bacteriocin, antimicrobial activity, low toxicity, antibacterial mechanism

## Abstract

Bacteriocins are a class of proteins produced by bacteria that are toxic to other bacteria. These bacteriocins play a role in bacterial competition by helping to inhibit potential competitors. In this study, we isolated and purified a novel bacteriocin Pkmh, different from the previously reported bacteriocin PA166, from *Pseudomonas* sp. strain 166 by ammonium sulfate precipitation, dialysis membrane method, ion exchange chromatography, and gel filtration chromatography. SDS-PAGE (sodium dodecyl sulfate–polyacrylamide gel electrophoresis) revealed that the molecular weight of Pkmh is approximately 35 kDa. Pkmh exhibited potent antimicrobial activity against bovine *Mannheimia haemolytica* (*M. haemolytica*) with low cytotoxicity, and lower hemolytic activity was observed. In addition, Pkmh retained antimicrobial activity at different pH ranges (2–10) and temperature conditions (40, 60, 80, 100 °C). Our analysis of its antimicrobial mechanism showed that Pkmh acts on bacterial cell membranes, increasing their permeability and leading to cell membrane rupture and death. In conclusion, Pkmh exhibited low hemolytic activity, low toxicity, and potent antibacterial effects, suggesting its potential as a promising candidate for clinical therapeutic drugs.

## 1. Introduction

Bovine respiratory disease remains one of the greatest challenges facing beef producers, veterinary inspectors, and feedlot managers. Although many factors play a role in the development of bovine respiratory disease, diseases caused by bacteria, particularly *M. haemolytica*, have long been a major impediment to cattle production overseas, causing economic losses of up to $300 million per year.

Current commercial vaccines used to prevent *M. haemolytica* do not provide complete protection offering only about 50% protection, so antibiotics remain an effective tool to control *M. haemolytica*. In recent years, it has become common to isolate *M. haemolytica* strains that are resistant to many different classes of antimicrobials [1]. Magstadt et al. found that more than 75% of *M. haemolytica* isolates from cattle treated with three or more antimicrobials were resistant to enrofloxacin, spectinomycin, tilmicosin, and tulathromycin [2]. Wettstein et al. found that 50% of 20 strains of *M. haemolytica* of bovine origin isolated from the respiratory tract of different animals were resistant to tetracycline, ampicillin, and streptomycin [3]. Therefore, there is an urgent need to research and develop new antimicrobial drugs with unique antimicrobial mechanisms.

Bacteriocins have received much attention from researchers as one of the potential alternatives to antibiotics. Bacteriocins are antimicrobial peptides or complex proteins synthesized by the ribosomes of bacteria and archaea. In addition to their potent bacterial inhibitory activity, bacteriocins have antifungal, antiviral, and even anticancer potential, and play an important role in regulating the balance of microbial communities and promoting plant growth [4]. The multi-functionality of bacteriocins has shown great promise in a variety of fields, including medicine, agriculture, and environmental health.

In the previous study, we found a strain of *Pseudomonas* sp. strain 166 with antimicrobial activity against *M. haemolytica* of bovine origin, and in this study, we optimize its culture conditions, purify its secreted bacteriocin, and elucidate the safety and mechanism of action of this bacteriocin with the aim of searching for a safe antibiotic alternative with strong antimicrobial activity.

## 2. Results

### 2.1. Optimization of Culture Conditions for Pkmh Production

In order to increase the yield of Pkmh, the culture conditions of *Pseudomonas* sp. strain 166 were optimized and the results are shown in Table 1. The experimental results showed that the active substance produced by this strain had the strongest antibacterial effect when a 500 mL Erlenmeyer flask was filled with 200 mL LB medium. At the same time, changes in the pH of the medium did not significantly affect the antimicrobial activity of Pkmh. However, temperature had a significant effect on the activity of Pkmh. Pkmh showed the highest antimicrobial activity when incubated at a low temperature of 16 degrees Celsius, while its antimicrobial activity was completely lost at a high temperature of 34 degrees Celsius. In addition, the synthesis of bacteriocin Pkmh started at 36 h of fermentation and peaked at 60 h of fermentation.

### 2.2. Purification of Pkmh from Pseudomonas sp. 166

**Figure 1:**
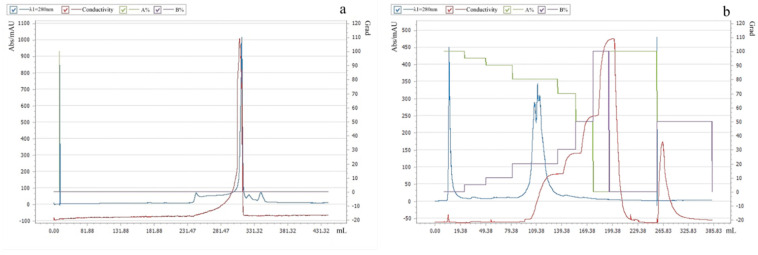
Purification of Pkmh: (**a**) PrePack 16/600 Finedex 75 prep grade purification; (**b**) Q-Sepharose column chromatography purification.

In this study, Pkmh was purified using PrePack 16/600 Finedex 75 prep grade and Q-Sepharose column chromatography. First, a crude bacteriocin Pkmh sample was applied to a PrePack 16/600 Finedex 75 prep grade. The active fractions were then further purified by stepwise elution using Q-Sepharose column chromatography. The purification procedure is shown in Figure 1.

### 2.3. Molecular Mass Determination of Pkmh

**Figure 2:**
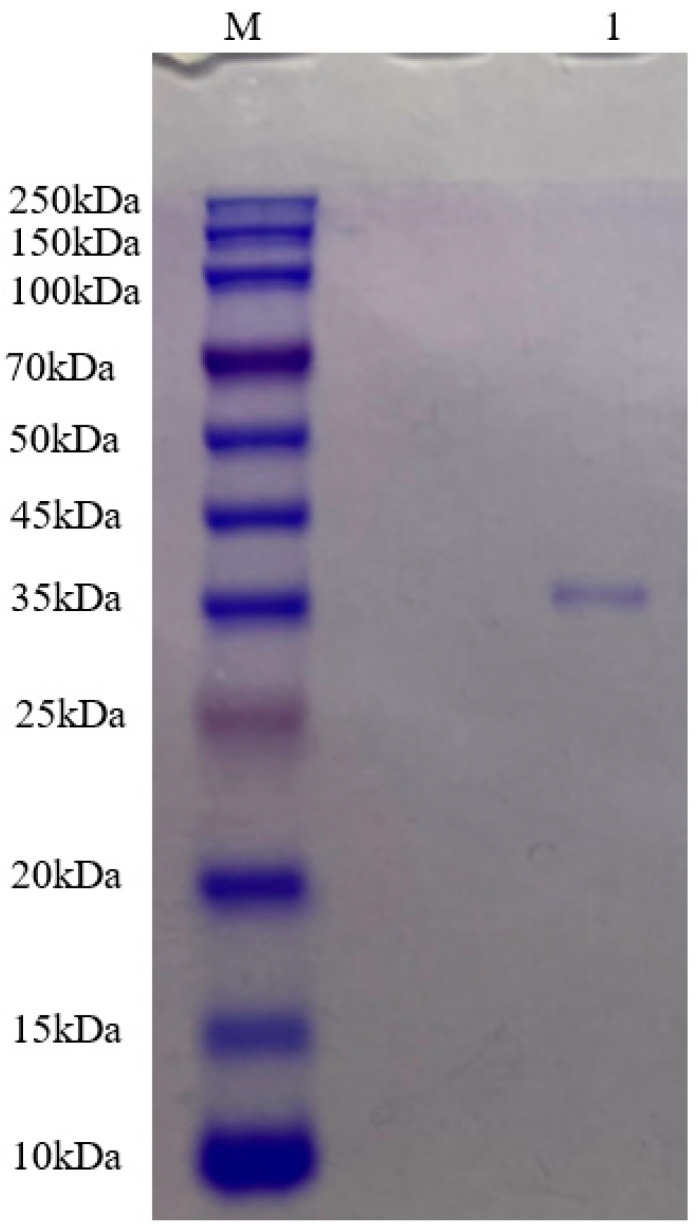
Molecular mass of Pkmh was analyzed by SDS-PAGE. M: rainbow marker; 1: Pkmh loaded on the gel.

The purified Pkmh was analyzed by SDS-PAGE. A single band with a molecular mass size of approximately 35 kDa was observed (Figure 2).

### 2.4. Stability of Pkmh

**Figure 3:**
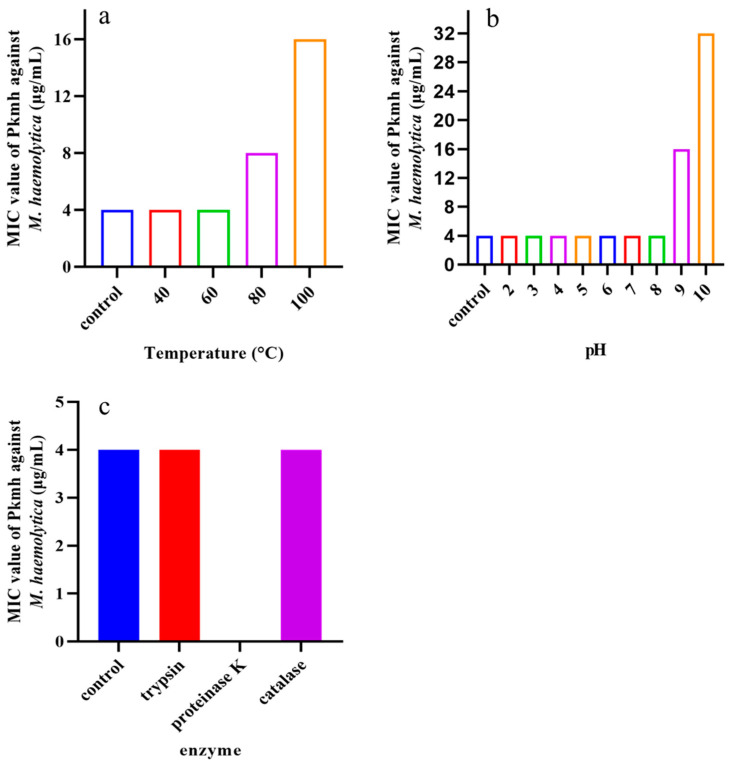
Stability analysis of Pkmh against temperature, pH, and enzyme. (**a**) Effect of temperatures on the stability of Pkmh; (**b**) effect of pH on the stability of Pkmh; (**c**) effect of enzymes on the stability of Pkmh.

Temperature, pH, and various enzymes on the antimicrobial activity of Pkmh were tested. As shown in Figure 3, the MIC was 16 μg/mL at pH 9 and increased to 32 μg/mL at pH 10. At high temperatures of 80 °C and 100 °C the MIC was 16 μg/mL and 32 μg/mL, respectively. In addition, the antimicrobial activity of Pkmh completely disappeared in the presence of proteinase K.

### 2.5. Antimicrobial Activity

**Figure 4:**
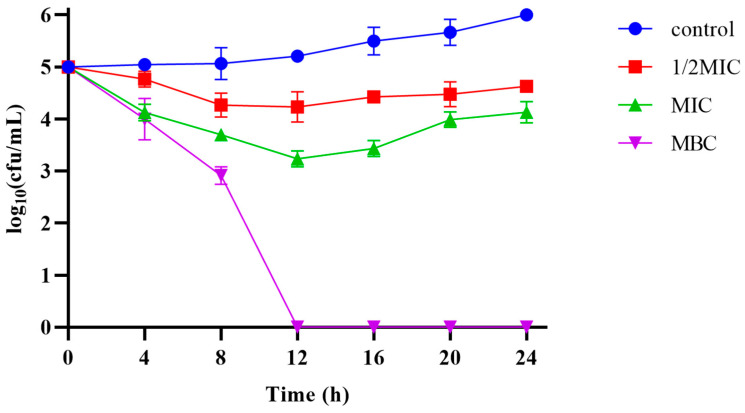
Time-kill kinetic curves of the Pkmh at 1/2MIC, 1×MIC, and MBC against *M. haemolytica*.

The MIC and MBC of Pkmh against *M. haemolytica* were 4 µg/mL and 12 µg/mL, respectively. The results of the bactericidal kinetic curves (Figure 4) showed that Pkmh was able to inhibit bacterial growth during the first 12 h at MIC and 1/2 MIC concentrations, while it was able to kill bacteria well at MBC concentrations compared to the control group.

### 2.6. Hemolytic Activity

**Figure 5:**
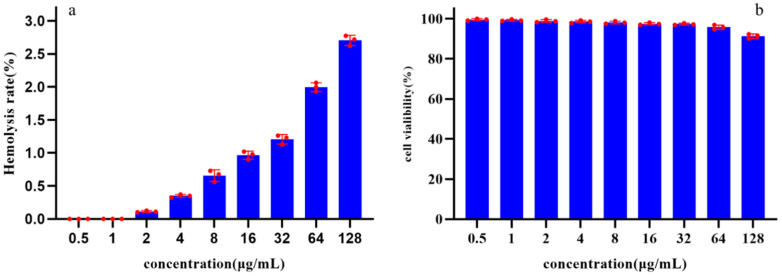
Safety analysis of Pkmh: (**a**) Hemolytic activities of Pkmh at different concentrations (0.5~128 μg/mL) on rabbit erythrocytes. (**b**) Cytotoxicity of Pkmh at different concentrations (0.5~128 μg/mL) on Vero cells.

The toxicity of Pkmh was evaluated by assessing its ability to cause lysis of rabbit red blood cells at concentrations ranging from 0.5 to 128 µg/mL. The results showed that the hemolysis rates of Pkmh against rabbit red blood cells were all less than 3% at concentrations ranging from 0.5 to 128 µg/mL (Figure 5a).

### 2.7. Cytotoxicity

The cytotoxicity of Pkmh was determined using the Cell Counting Kit-8. After 24 h of incubation, the cell viability results shown in Figure 5b indicate that Pkmh does not exhibit significant cytotoxicity towards Vero cells. Even at a concentration of 128 µg/mL, the survival rate of Vero cells remains above 90%.

### 2.8. Cytoplasmic Membrane Permeability 

**Figure 6:**
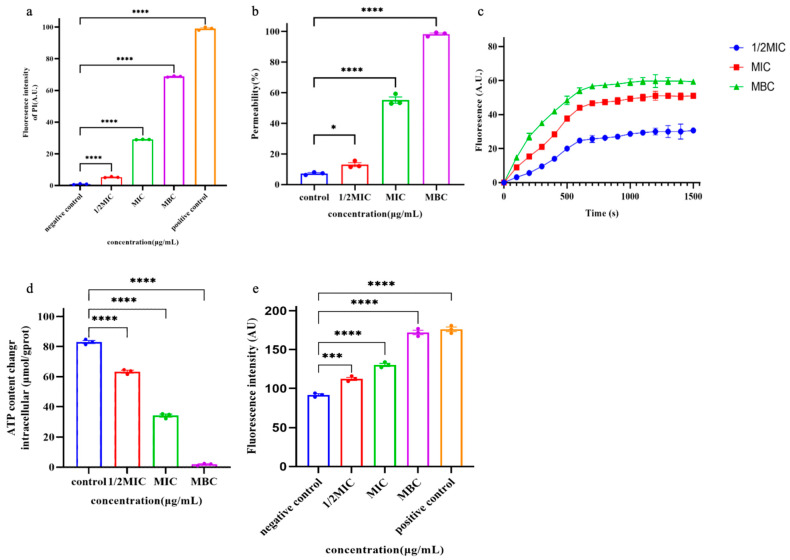
Studies on the mechanism of action of Pkmh. (**a**) Cytoplasmic membrane permeability of the Pkmh. *M. haemolytica* was treated with different concentrations of Pkmh and the changes in fluorescence intensity of PI in the bacterial cells were monitored at an excitation wavelength of 615 nm and an emission wavelength of 535 nm. (**b**) Outer membrane permeabilization assays of Pkmh. NPN uptake by *M. haemolytica* was determined using the NPN assay at different concentrations of Pkmh. NPN uptake was monitored at an excitation wavelength of 350 nm and an emission wavelength of 420 nm. (**c**) Cytoplasmic membrane permeability of Pkmh. Changes in the cytoplasmic membrane potential of *M. haemolytica* treated with different concentrations of Pkmh were assessed by the release of the membrane potential-sensitive dye DiSC3-5. The changes in fluorescence intensity over time were monitored at an excitation wavelength of 622 nm and an emission wavelength of 670 nm. (**d**) Effect of Pkmh on intracellular ATP levels in *M. haemolytica*. (**e**) Effect of Pkmh on intracellular ROS levels in *M. haemolytica*. Data for (**a**,**b**,**d**,**e**) are expressed as mean ± SEM. * *p* < 0.05, *** *p* < 0.001, and **** *p* < 0.0001, Tukey’s multiple comparison test for significant differences compared with controls.

PI is a small fluorescent molecule and the uptake and rejection of PI can be used to distinguish between dead cells and living cells with intact cell membranes [5]. The interaction between Pkmh and bacterial cell membranes was further investigated by measuring the uptake of PI into bacterial cells. As shown in Figure 6a, Pkmh penetrated the cytoplasmic membrane in a concentration-dependent manner.

### 2.9. Outer Membrane Permeability Assay

Normally, the hydrophobic fluorescent substance NPN is excluded by the outer membrane; however, when the permeability of the outer membrane is increased, NPN is taken up, leading to an increase in the fluorescence intensity of the cells [6]. Therefore, the outer cell membrane permeabilizing activity of Pkmh was assessed by measuring the uptake of NPN by bacterial strains. As shown in Figure 6b, Pkmh permeated the outer membrane in a concentration-dependent manner at concentrations ranging from 0.25 to 128 μg/mL. When the concentration of Pkmh exceeds 0.5 µg/mL, the permeability of the bacterial outer membrane induced by Pkmh exceeds 80%.

### 2.10. Membrane Depolarization

In this study, we investigated the effect of Pkmh on the plasma membrane potential of *M. haemolytica* using the dye DiSC_3_-5. Upon membrane depolarization, the DiSC_3_-5 dye accumulates on the bacterial membrane in a quenched state and is accessible to the solution, leading to an increase in its fluorescence intensity. As shown in Figure 6c, Pkmh induced a sustained release of diSC_3_-5 over 1500 s and showed a dose-dependent effect.

### 2.11. Release of ATP and ROS

As shown in Figure 6d, we observed a decrease in intracellular ATP in *M. haemolytica* cells that correlated with the dose of Pkmh. In addition, Pkmh induced the accumulation of ROS as shown in Figure 6e. This in turn increased membrane damage, resulting in a more pronounced impairment of internal bacterial homeostasis.

## 3. Discussion

Environmental factors such as temperature, pH, and the composition of the growth medium can affect the amount of bacteriocin produced [7]. According to Trinetta et al., the ability of lactic acid bacteria (LAB) to produce bacteriocins is dependent on their growth, with maximum activity usually coinciding with the time when cell growth is at its maximum [8]. In our previous study, we found that the inhibition diameter of *Pseudomonas* sp. 166 against *P. multocida* of bovine origin could reach about 40 mm at a temperature of 16 °C and an incubation time of 60 h, but only 32 mm for *M. haemolytica* [9]. Therefore, we optimized the culture conditions of *Pseudomonas* sp. strain 166 to increase the yield and activity of Pkmh. The antimicrobial activity of Pkmh was found to be affected by medium aeration, incubation temperature, and incubation time. However, surprisingly, changes in the pH of the medium did not significantly affect the antimicrobial activity of Pkmh, in agreement with the findings of Ahmed et al. [10]. Interestingly, the synthesis of Pkmh was significantly affected by temperature, with the synthesis of Pkmh decreasing with increasing temperature until it stopped completely at 34 °C. This phenomenon may be explained by the fact that *Pseudomonas* sp. strain 166 has evolved a unique mechanism for survival and reproduction in cold environments, centered on the synthesis of Pkmh to counteract competition with other microorganisms at low temperatures. Our results are consistent with Benaud’s findings, which highlight the crucial role of secondary metabolite production as a central component of microbial survival strategies in the face of extreme environments [11]. This suggests that under cold and nutrient-poor conditions, Pkmh production is not only crucial for *Pseudomonas* sp. strain 166 to withstand external challenges, but also an effective means for it to maintain its ecological niche and ensure the persistence of the community.

The purification of bacteriocins is an essential step in the in-depth study of the properties and functions of these compounds. Ammonium sulfate precipitation, dialysis membrane, macroporous resin column, ion exchange chromatography, and gel chromatography are commonly used methods for the purification of bacteriocins [12,13]. In this study, ammonium sulfate precipitation, dialysis membrane method, ion exchange chromatography, and gel filtration chromatography were used for the purification of Pkmh. The molecular weight of Pkmh was then determined by SDS-PAGE to be approximately 35 kDa. The stability of bacteriocins is one of the most important indicators of their potential use. Although bacteriocins often show resistance to heat, it is important to note that this property is not uniformly present in all types [14]. In this study, the antimicrobial activity of Pkmh responded differently to changes in temperature, particularly at 80 °C and 100 °C, with a slight decrease in antimicrobial activity, which is different from the stability of bacteriocin PA166 secreted by *Pseudomonas* sp. 166 [15]. Pkmh exhibited stable antimicrobial activity over a wide range of pH from 2 to 8, but the antimicrobial activity was slightly reduced when the ambient pH was higher than 9, highlighting its adaptability in acidic to slightly alkaline environments. The stabilization of Pkmh in the low pH environment provides the feasibility for direct oral administration. In contrast, in the high pH environment of the small intestine, the solubility and permeability of Pkmh and its antimicrobial activity may be compromised, affecting its in vivo distribution and efficacy [16]. Nevertheless, the overall stability of Pkmh provides a solid basis for its use in clinical applications.

Several bacteriocins have favorable properties, such as low MIC values, low immunogenicity, toxicity, and resistance induction potential, which have prompted investigations into their potential clinical applications [17,18,19,20]. The first step in evaluating the biological efficacy of bacteriocins against clinically relevant bacterial pathogens is to perform in vitro antimicrobial activity assays [21]. Bacteriocin PA996 produced by *Pseudomonas azotoformans* has a MIC of 16 μg/mL against *Pasterella multocida* [9]. The MIC of a novel bacteriocin from *Pseudomonas aeruginosa* 43 against methicillin-resistant *Staphylococcus aureus* [22]. In this study, the MIC and MBC of Pkmh against *M. haemolytica* were 4 µg/mL and 12 µg/mL, respectively. In addition, Pkmh significantly inhibited the growth of *M. haemolytica* within the first 12 h at the MBC concentration. This suggests that it may be a potential candidate for the treatment of *M. haemolytica* infections. The low toxicity of bacteriocins results in minimal adverse effects on the host during the treatment process, a characteristic that contrasts sharply with the side effects associated with many traditional antibiotics [23,24]. Some studies have shown that at active concentrations, bacteriocins have little cytotoxic effect on a variety of mammalian cell lines [16]. We evaluated the hemolysis of rabbit erythrocytes and the cytotoxicity of Pkmh on Vero cells. At a Pkmh concentration of 128 µg/mL, Vero cell survival was over 90% and erythrocyte hemolysis was less than 3%. Pkmh has shown an improved safety profile compared to bacteriocin PA166 [15] and the favorable safety profile of Pkmh is a key factor for its use in healthcare.

Various modes of action have been proposed and identified for bacteriocins, such as pore formation and inhibition of cell wall/nucleic acid/protein synthesis [25]. The most common antimicrobial mechanism of bacteriocins is cell membrane disruption, binding to specific receptor proteins on the cell membrane and forming pores that increase cell membrane permeability, leading to cell death [12,26]. In this study, we investigated the mechanism of action of Pkmh on bacteria using an outer membrane permeability assay and cytoplasmic membrane permeability and showed that bacteriocins kill bacteria by disrupting cell membranes. Disruption of cell membrane integrity by Pkmh resulted in membrane depolarization, ATP release, and ROS accumulation. The disruption of cellular membrane integrity leads to leakage of ATP to the outside of the cell, and when intracellular ATP levels are low and ions are deficient, the production of DNA, RNA, proteins, and polysaccharides is inhibited [27,28]. ROS are involved in numerous processes within cells in different domains of life, including bacteria, plants, and animals, including humans [29]. In addition, it is now believed that cell death is actually the result of ROS triggering physiological or programmed cell death pathways [30]. The results reveal the process by which Pkmh induces cell death by disrupting cell membrane structure, which in turn affects intracellular energy metabolism and redox homeostasis, and ultimately induces cell death. Pkmh has great potential in the treatment of infectious diseases caused by *M. haemolytica*. This discovery has far-reaching and important implications for the exploration of novel antimicrobial strategies and the development of innovative drugs.

## 4. Materials and Methods

### 4.1. Materials

Mueller–Hinton agar (MH), LB broth, ammonium sulfate, cut-off membrane, and fetal bovine serum were purchased from Sangon Biotech (Shanghai, China) Co., Ltd. The ATP assay kit, reactive oxygen species (ROS) assay kit, dipropylthiadicarbocyanine (diSC_3_-5), propidium iodide (PI), and N-phenyl-1-naphthylamine (NPN) were purchased from Sigma-Aldrich (Shanghai, China). Q-μSphere column was purchased from Wuxi Tianyan Biotechnology Co., Ltd. PrePack 16/600 Finedex 75 pg was purchased from Jiaxing Qianchun Biotech Co., Ltd. (Jiaxing Qianchun, Zhengjiang, China).

### 4.2. Bacterial Strains and Growth Conditions

*M. haemolytica* and *Pseudomonas* sp. strain 166 were provided by the Laboratory of Pharmacology, Jilin Agricultural University, Jilin, China. *Pseudomonas* sp. strain 166 was used for the production of bacteriocin and was cultured in an LB medium. *M. haemolytica* was used as an indicator organism and cultured in MH agar (containing 5% fetal bovine serum) at 37 °C for 18 h.

### 4.3. Optimization of Culture Conditions for Pseudomonas sp. Strain 166 Fermentation

The activity of Pkmh is defined as the reciprocal of the minimum dilution that results in the formation of an inhibition zone around the indicator bacterial lawn and is expressed in units of activity (AU) per mm (AU mL^−1^ = reciprocal of the highest twofold dilution × 100) [31]. We investigated the effect of different medium aeration, medium pH, incubation temperature, and incubation time on Pkmh activity units.

#### 4.3.1. Effect of Media Aeration on Pkmh Activity Units

To study the effect of aeration on Pkmh synthesis, different volumes of LB medium were added to 500 mL Erlenmeyer flasks and incubated at 16 °C, 100 rpm/min for 72 h. After incubation, Pkmh activity units were measured by the agar well diffusion method.

#### 4.3.2. Effect of Medium pH on Antimicrobial Activity

The pH of the medium was adjusted to 5.5, 6.5, and 7.5, 1% of the *Pseudomonas* sp. strain 166 was inoculated into 200 mL of LB medium and incubated at 16 °C and 100 rpm/min for 72 h. After incubation, Pkmh activity units were measured by the agar well diffusion method.

#### 4.3.3. Effect of Different Incubation Temperatures on Antimicrobial Activity

One hundred percent *Pseudomonas* sp. strain 166 was accessed in 200 mL of LB medium and the incubation temperature was added to 200 mL of LB medium and incubated for 72 h at 10 °C, 16 °C, 22 °C, 28 °C, and 34 °C at 100 rpm. After incubation, Pkmh activity units were measured by the agar well diffusion method.

#### 4.3.4. Effect of Different Incubation Times on Pkmh Activity

In this study, 1% *Pseudomonas* sp. strain 166 was accessed in 200 mL of LB medium, inoculated into LB medium, and incubated at 100 rpm/min at 16 °C for 24 h, 36 h, 48 h, 60 h, and 72 h. After incubation, the Pkmh activity units were measured by the agar well diffusion method.

### 4.4. Purification of Bacteriocin Pkmh from Pseudomonas sp. Strain 166

#### 4.4.1. Preparation of Crude Extract

The crude extract was prepared according to a previous study [32]. Briefly, *Pseudomonas* sp. strain 166 was grown in LB broth at 16 °C for 60 h. The supernatant was centrifuged at 8000× *g* for 30 min at 4 °C, then ammonium sulfate was added to the supernatant, and the mixture was stirred overnight at 4 °C. The precipitate was then collected and dissolved in phosphate-buffered saline. The crude bacteriocins were concentrated. Bacteriostatic activity was determined by the agar well diffusion method.

#### 4.4.2. Purification of Pkmh

The crude bacteriocin was purified using the ClearFirst-3000 Protein Purification System (Flash, Shanghai, China). Five millimeters of the crude sample were injected through PrePack 16/600 Finedex 75 prep grade and equilibrated with PBS at a flow rate of 0.5 mL/min. The eluted fractions were analyzed at 280 nm and the fractions were collected consecutively in units of 1 mL. Antibacterial activity against *M. haemolytica* was determined by the agar diffusion method.

The active fractions were desalted by dialysis using a 5-kDa cut-off membrane at 4 °C. The active fractions were then further purified on a Q-μSphere column using the ClearFirst-3000 Protein Purification System. Prior to purification, the column was pre-equilibrated with deionized water at a flow rate of 1 mL/min. During the purification process, solutions containing different concentrations of sodium chloride (100 mM, 200 mM, 300 mM, and 400 mM, respectively) were used as eluents and the flow rate was again maintained at 1 mL/min. The eluted fractions were analyzed at 280 nm; the active fractions were dialyzed again at 4 °C using a 5 kDa cut-off membrane to further remove residual small molecules, and then concentrated using a rotary evaporator (Yingdi, Shanghai, China). Finally, the antibacterial activity against *M. haemolytica* was determined by the agar diffusion method. The samples were then subjected to molecular mass determination by SDS-PAGE.

### 4.5. Stability Assays

The stability of Pkmh was determined as previously described [33]. To determine the thermal stability, Pkmh was heated at temperatures of 40 °C, 60 °C, 80 °C, and 100 °C for 30 min and then cooled in an ice bath.

The pH sensitivity of Pkmh was adjusted to 2~10 using 1M HCl and 1M NaOH, and each mixture was incubated at room temperature for 2 h and then neutralized.

The effect of Pkmh on various enzymes was determined using trypsin, proteinase K and catalase at 37 °C for 1 h, the final concentration of these enzymes is 1 mg/mL.

Untreated bacteriocin Pkmh was used as a control, and all samples were tested for residual antibacterial activity using the agar diffusion assay. 

### 4.6. Determination of Antimicrobial Activity of Pkmh

The antibacterial activity of bacteriocin Pkmh was determined by the minimum inhibitory concentration (MIC) against *M. haemolytica*. In total, 100 μL of a twofold serial dilution of bacteriocin Pkmh was added to microtiter plates, followed by 100 μL of *M. haemolytica* cell suspension to a final concentration of 10^6^ CFU/mL. The microplates were then incubated at 37 °C for 12 h and the absorbance at 600 nm was determined using a Multi-Function Enzyme Labeler (TECAN, Männedorf, Switzerland). The minimum bactericidal concentration (MBC) is defined as the lowest concentration of a Pkmh capable of killing 99.99% of bacteria. To determine the MBC, 100 µL of the sample from the well containing the MIC and the three preceding wells are transferred to the MHA solid medium. The plates are then incubated overnight at 37 °C before colony counting. 

The time-kill kinetic curve assay was then performed. The concentration of *M. haemolytica* was adjusted to 10^6^ CFU/mL and mixed with MBC, 1×MIC, and 1/2MIC final concentrations of Pkmh. *M. haemolytica* cell suspension with PBS was used as a control. Cultures were coated with MH agar (containing 5% fetal bovine serum) every 4 h and incubated at 37 °C for 18 h to count the total number of viable cells.

### 4.7. Measurement of Hemolytic Activity

The hemolytic activity of Pkmh was tested according to a previous study [34]. In brief, healthy rabbit blood cells were washed three times with PBS by centrifugation at 1000 g for 5 min and the final concentration was adjusted to 1% with PBS. Bacteriocin Pkmh at a final concentration of 0.5–128 μg/mL was mixed with the blood cells, and Triton X-100 (0.2%) and PBS were used as positive and blank controls, respectively. The absorbance at 576 nm of the released hemoglobin was measured using a Multi-Function Enzyme Labeler. The percentage of hemolysis was then calculated. 

### 4.8. Determination of Cytotoxicity (CCK8 Assay)

Vero cells were used for cytotoxicity studies as previously described [9]. Cells were cultured in 96-well microtiter plates at 5000 cells per well for 36 h. Pkmh was added at a final concentration of 0.5~128 μg/mL, and the medium and cells without added Pkmh were used as control and blank control groups, respectively. After 24 h of incubation, 10 μL CCK-8 solution was added and incubated for 2 h. The optical density at 450 nm was measured using a Multi-Function Enzyme Labeler.

### 4.9. Cytoplasmic Membrane Permeability

*M. haemolytica* was cultured overnight at 37 °C in MH (containing 5% fetal bovine serum) and centrifuged at 5000× *g* for 10 min at 4 °C. The cells were washed three times with PBS and resuspended in MH to 10^6^ CFU/mL. A final concentration of 10 μM propidium iodide (PI) was added to the bacterial suspension. Pkmh was then added to the bacterial suspension until its concentration reached 1/2 MIC, MIC, and MBC, respectively, while an untreated cell suspension was used as a negative control and a 1% Triton X-100-treated group as a positive control. Finally, fluorescence intensity was measured using an F4500 fluorescence spectrophotometer at an excitation wavelength of 615 nm and an emission wavelength of 535 nm.

### 4.10. Outer Membrane Permeability Assay

NPN was used to measure bacterial outer membrane permeability as previously described [35]. Briefly, bacterial suspensions were incubated with a final concentration of 10 μM NPN for 30 min and then incubated with Pkmh (1/2 MIC, MIC, and MBC) for 1 h. Fluorescence was defined using an F4500 fluorescence spectrophotometer (excitation λ = 350 nm, emission λ = 420 nm).

### 4.11. Membrane Depolarization

The effect on cell membrane potential was determined using the fluorescent dye diSC_3_-5, which is sensitive to cell membrane potential. *M. haemolytica* grown to mid-log phase was washed 3 times with 5 mM HEPES buffer and the OD600 was adjusted to 0.5, then diSC_3_-5 was added and the mixture incubated at 37 °C for 20 min. Pkmh of 1/2MIC, MIC, and MBC were added to the mixture, followed by the detection of fluorescence changes from 0 to 1500 s using an F4500 fluorescence spectrophotometer (excitation wavelength λ = 622 nm, emission wavelength λ = 670 nm).

### 4.12. Measurement of Reactive Oxygen Species (ROS)

ROS levels in *M. haemolytica* treated with Pkmh (1/2MIC, MIC and MBC) were measured using a reactive oxygen species assay kit. In short, a final concentration of 10 μM of the fluorescent probe DCFH-DA was added to the bacterial suspension and incubated at 37 °C for 30 min, washed three times with PBS. The bacteria were treated with bacteriocin Pkmh and incubated at 37 °C for 1 h. Fluorescence measurements were performed using an F4500 fluorescence spectrophotometer (excitation λ = 350 nm, emission λ = 420 nm).

### 4.13. Measurement of Adenosine 5′-Triphosphate (ATP)

ATP levels in *M. haemolytica* were determined using an ATP test kit. After treatment with bacteriocin Pkmh for 4 h, the mixtures were centrifuged at 10,000× *g* for 10 min at 4 °C and the cells were collected. Cells were lysed with pre-cooled lysozyme, centrifuged, and the supernatant collected. Intracellular ATP levels were determined using a Multi-Function Enzyme Labeler.

### 4.14. Statistical Analysis

Data were analyzed by ANOVA using GraphPad Prism 9.0, and significant differences between means were assessed by Tukey’s multiple comparison test. Quantitative data were expressed as mean ± standard error of the mean (SEM). * *p* < 0.05, ** *p* < 0.01, *** *p* < 0.001, and **** *p* < 0.0001.

## 5. Conclusions

In this study, we successfully purified the bacteriocin Pkmh from *Pseudomonas* sp. 166, determined its molecular weight to be 35 kDa, and were able to confirm that Pkmh belongs to the class of proteins based on its sensitivity to proteinase K. In addition, Pkmh showed satisfactory stability and safety. In addition, Pkmh showed satisfactory stability and safety, properties that pave the way for its biomedical applications. Of particular note is the remarkable antimicrobial activity of Pkmh against *M. haemolytica*, which disrupts the membrane structure of the target cell, leading to irreversible damage and death of the microbial cell. Subsequent research programs will be devoted to deciphering the chemical structure and formula of Pkmh in order to fully elucidate its unique principle of bioactivity and mode of action.

## Figures and Tables

**Table 1:** Evaluation of bacteriocin production under different conditions.

	Conditions	Activity (AU/mL)
Volume (mL)	100	800
	200	3200
	300	1600
Medium pH	5.5	3200
	6.5	3200
	7.5	3200
Temperature (°C)	10	-
	16	6400
	22	3200
	28	800
	34	-
Incubation times (h)	24	-
	36	200
	48	1600
	60	6400
	72	6400

(-) not detected.

## Data Availability

The datasets and materials used and/or analyzed during the current study are available from the corresponding author upon reasonable request.
